# Listeriosis at a Tertiary Care Hospital in Beijing, China: High Prevalence of Nonclustered Healthcare-Associated Cases Among Adult Patients

**DOI:** 10.1093/cid/cis943

**Published:** 2012-11-21

**Authors:** Huan-ling Wang, Khalil G. Ghanem, Peng Wang, Shuang Yang, Tai-sheng Li

**Affiliations:** 1 Department of Infectious Diseases, Peking Union Medical College Hospital, Chinese Academy of Medical Sciences, Beijing, People's Republic of China; 2 Department of Medicine, Division of Infectious Diseases, Johns Hopkins University School of Medicine, Baltimore, Maryland; 3 Department of Microbiology, Peking Union Medical College Hospital; 4 Department of Medicine, PekingUnion Medical College; 5 Department of Infectious Diseases, Peking Union Medical College Hospital, Chinese Academy of Medical Sciences, Beijing, People's Republic of China

**Keywords:** *Listeria monocytogenes*, immunocompromised host, healthcare-associated infection, neonatal, maternal

## Abstract

***Background.*** Listeriosis is an emerging infectious disease associated with high mortality. There are few published reports from East Asia and developing countries. Our goal was to describe the clinical characteristics and outcomes of patients diagnosed with *Listeria monocytogenes* at a tertiary care hospital in Beijing, China.

***Methods.*** Peking Union Medical College Hospital (PUMCH), an 1800-bed hospital, consists of 2 campuses that house different medical departments. We retrospectively reviewed all culture-proven cases of listeriosis occurring at PUMCH between 1999 and 2011. Point estimates and 95% confidence intervals are presented.

***Results.*** There were 38 patients with listeriosis: 5 neonatal, 8 maternal, and 25 nonmaternal. The median age of the adult nonmaternal patients was 47 (range, 18–79) years with a female predominance (72%). Forty percent (n = 10) had an underlying rheumatic disease. Forty-four percent of cases (n = 11) were healthcare-associated infections occurring a median of 20 (range, 3–44) days after hospital admission. Only 2 of the 11 healthcare-associated cases clustered in space and time. One healthcare-associated case occurred in a patient receiving KHI-272 therapy, an oral, irreversible dual EGFR/HER2 inhibitor. The neonatal and maternal listeriosis cases were similar to those reported in the literature.

***Conclusions.*** Nonclustered healthcare-associated cases of *L. monocytogenes* occurred at a large tertiary care hospital in Beijing, China. The source of these infections is unclear. Although rare, in the setting of immunosuppression, *Listeria* should be considered in the differential diagnosis of healthcare-associated infections, even in the absence of a point-source outbreak.

## Abstract (precis)

Thirty-eight cases of listeriosis over a 12-year period from inpatients at a tertiary care hospital in China were reviewed. We found a high prevalence of healthcare-associated cases that did not cluster in time and space.

Listeriosis is a relatively uncommon but serious infection caused by *Listeria monocytogenes*. This organism is ubiquitous in the environment and can survive at temperatures ranging from −7°C to body temperature [1]. The main route of transmission is believed to be through the consumption of contaminated food (processed meats, unpasteurized milk, soft cheeses, and cantaloupes) [2–7] and vertical transmission from mother to child [8, 9]. However, healthcare-associated transmission has also been reported through patient-to-patient transmission, mineral bathing oil, contaminated resuscitation equipment, and the contaminated hands of medical personnel [10–14]. Most of the healthcare-associated infections are clustered and related to food processing [11–13].

Gastroenteritis, bacteremia, and meningitis are the most common manifestations of listeriosis. Because *L. monocytogenes* has a strong predilection for elderly and immunocompromised persons [15–18], results in poor fetal outcomes [19–21], exhibits poor response to third-generation cephalosporins, and is associated with a high mortality rate, it has become an increasingly important emerging infectious disease [22].

In the United States, *L. monocytogenes* is the fourth causative microorganism of bacterial meningitis [23]. Among persons aged >65 years, *L. monocytogenes* is the third leading pathogen [24, 25]. Most listeriosis cases have been reported from industrialized Western countries. Reports from East Asia and developing countries are scarce [26, 27].

Our goal was to retrospectively review all culture-proven cases of listeriosis at Peking Union Medical College Hospital (PUMCH) since 1999 and describe the clinical characteristics and outcomes of the infected patients.

## METHODS

PUMCH is an 1800-bed tertiary care hospital in Beijing, China. Founded in 1921 by the Rockefeller Foundation, PUMCH is the national medical technical support center for the diagnosis and treatment of severe and complicated diseases. In 2002, another hospital in Beijing merged with PUMCH and was renamed the Western campus of PUMCH. The latter housed several departments (general medicine, rheumatology, oncology, and breast surgery), and both campuses shared other departments (hematology, gastroenterology). PUMCH provides medical services to patients from surrounding areas (Beijing, and Hebei province) and to patients being referred from various outside institutions throughout China.

We retrospectively identified all patients with *L. monocytogenes* infections based on a list generated from an electronic database in the clinical microbiology laboratory at PUMCH. All positive culture results for *L. monocytogenes* diagnosed at PUMCH since 1999 are stored in the database. We included all cases from January 1999 to October 2011. Clinical data from the identified cases were abstracted from the medical records. These data included demographic characteristics, comorbidities, known risk factors (immunosuppressive therapy, dietary history, travel, and exposures), the sites from which the organism was isolated, clinical presentation, laboratory data, type of antimicrobial therapy, duration of hospitalization, and outcomes.

The diagnosis of listeriosis was based on one of the following: isolation of *L. monocytogenes* from normally sterile clinical specimens (eg, cerebrospinal fluid [CSF], blood, amniotic fluid, uterine swab); isolation of *L. monocytogenes* from nonsterile specimens (eg, rectal swab, tracheal swab); and histopathology compatible with listeriosis [22]. Cases were categorized as neonatal, maternal, or nonmaternal infections. All maternal cases were in pregnant women who had *L. monocytogenes* isolated from cultures of normal sterile body sites or vaginal swab [19]. Healthcare-associated cases were defined as onset of listeriosis symptoms >48 hours after admission for medical conditions other than listeriosis.

We used descriptive statistics. Where appropriate, we present point estimates with 95% confidence intervals (CIs). This study was reviewed and approved by the Institutional Review Board at PUMCH.

## RESULTS

We identified 38 patients (cases) of listeriosis diagnosed between 1999 and 2011. The demographic characteristics of these cases are summarized in Table 1. There were 5 neonatal, 8 maternal, and 25 nonmaternal infections with *L. monocytogenes*.

**Table 1.** Characteristics of 38 Cases of Listeriosis

Group	Neonatal	Maternal	Nonmaternal
Total	5 (13.2)	8 (21.1)	25 (65.8)
Male	4 (80)	0	9 (36)
Median age (min, max), y	NA	30 (26, 33)	47 (18, 79)
Median gestation (min, max), wk	37 (27, 39.9)	29 (18.9, 39.9)	NA
Underlying disease			23 (92)
Autoimmune disease		1 (12.5)	10 (40)
Neoplasm			10 (40)
Diabetes			3 (12)
Ulcerative colitis			2 (8)
Polycystic kidney and hepatic disease			1 (4)
Iatrogenic factors			
Chronic use of corticosteroids			10 (40)^a^
Chemotherapy			5 (20)
Clinical manifestations			
Fever	4 (80)	6 (75)	24 (96)
Gastrointestinal symptoms		5 (62.5)	12 (48)
Neurological symptoms		1 (12.5)	16 (68)
Laboratory findings			
Peripheral WBC, mean ± SD, 10^9^/L	13.3 ± 5.1	17.6 ± 6.2	8.3 ± 5.1
CSF WBC median (min, max), cells/µL	1660 (16, 128 300)		200 (36, 2590)
CSF neutrophils, %, median (min, max)	65 (62, 97)		40 (10, 96)
CSF mononuclear, %, median (min, max)	35 (3, 38)		60 (4, 90)
CSF neutrophils >50%	3/3 (100)		7/15 (46.7)
CSF protein median (min, max), g/L	1.8 (0.94, 9.16)		1.77 (0.65, 8.45)
Mortality	1 (20)	0	9 (36)

Data are presented as No. (%) unless otherwise specified.

Abbreviations: CSF, cerebrospinal fluid; max, maximum; min, minimum; NA, not applicable; SD, standard deviation; WBC, white blood cell.

^a^ On steroid of prednisone equivalent 30–40 mg/d in 4 of 10 cases, >50 mg/d in 6 of 10 cases; of those, 4 patients were on concurrent immunosuppressive therapies.

### Neonatal Listeriosis

Of 26 221 deliveries during this time period, there were 5 cases of neonatal listeriosis identified. Four of 5 cases of neonatal listeriosis were male. All 5 neonatal listeriosis cases were born to symptomatic mothers. All had positive cultures and presented with fetal distress (n = 5), sepsis (n = 4), meningitis (n = 4), Apgar score <5 (n = 3), low birth weight (n = 2), and meconium aspiration (n = 1), suggestive of intrauterine infection. The clinical characteristics and outcomes of these 5 cases are summarized in Table 2.

**Table 2.** Characteristics of 5 Neonatal Cases of Listeriosis

No.	Sex	Presentation	Maternal Illness	Gestation (wk)	Culture Sites	Initial Antibiotic	Switch Antibiotic	Intubation	Complication	Outcome
16	F	Fetal distress, Apgar 9, T_max_ 37.5°C, low birth weight, WBC 18.3 × 10^9^/L, S_p_O_2_ 76% on ambient air	High fever; positive cultures	37.1	Blood, rectal swab, laryngeal swab	Meropenem + PNG	No	No	Sepsis, meningitis, aspiration pneumonia, bilateral IVH	Survived
24	M	Fetal distress, C-section, SOB, Apgar 5, afebrile, WBC 15 × 10^9^/L, increased ICP, turbid CSF, CSF WBC 1660/µL	Diarrhea, fevers; positive cultures	31	Blood, rectal swab	Meropenem	PNG	Yes	Sepsis, meningitis, pneumonia, low birth weight, ICH	Survived
25	M	Fever (38°C), Apgar 1, SOB, cyanosis, rash, hypotension, WBC 16 × 10^9^/L, bloody and turbid CSF, CSF WBC 128 300/µL	Headache, fevers, severe abdominal pain. No microbiologic data.	32.7	Blood, laryngeal swab, tracheal tube tip	Cefmetazole	Meropenem + PNG	Yes	Sepsis, meningitis, pneumonia NRDS, Bilateral IVH, SAH	Survived
36	M	Fetal distress, Apgar 9, C-section, meconium aspiration, low fever (37.9°C), WBC 5.39 × 10^9^/L, CSF WBC 0	High fevers; positive cultures	39.9	Blood, laryngeal swab, tracheal tube tip	Meropenem	Ampicillin/sulbactam + cefepime	Yes	Sepsis, IVH	Survived
28	M	Extremely low birth weight (720 g), Apgar 5, WBC 11.4 × 10^9^/L	SLE, prednisone 10 mg/d, abdominal pain, no cultures placental pathology: acute chorioamnionitis	27	Rectal swab	Ampicillin/sulbactam	Meropenem	Yes	Intrauterine infection, pulmonary hemorrhage (NRDS), neonatal asphyxia, premature birth, extremely low birth weight, sclerema neonatorum	Deceased day 2

Abbreviations: C-section, cesarean section; CSF WBC, white blood cell count in cerebrospinal fluid; ICH, intracranial hemorrhage; ICP, intracranial pressure; IVH, intraventricular hemorrhage; NRDS, neonatal respiratory distress syndrome; PNG, penicillin; SAH, subarachnoid hemorrhage; SLE, systemic lupus erythematosus; S_p_O_2_, oxygen saturation from pulse oximetry; SOB, shortness of breath; T_max_, maximal temperature; WBC, peripheral white blood cell count.

### Maternal Listeriosis

There were 8 maternal cases of listeriosis identified. Six cases were confirmed by culture. Two other cases were suspected based on symptoms and positive cultures in their infants at the time of delivery. The median age was 30 years (range, 26–33 years). The median gestation was 29 weeks (range, 18.9–39.9 weeks). Maternal cases presented with a sudden onset (<1 week from presentation) of symptoms (n = 7), which included high fevers with a maximal temperature >39°C (n = 6), gastrointestinal symptoms (diarrhea, abdominal pain; n = 5), and various obstetrical manifestations (decreased fetal movement in 2 cases, intrauterine fetal death, vaginal bleeding, and acute pyelonephritis) (Table 3). Two maternal cases had *L. monocytogenes* cultured from blood; all 3 cases whose *L. monocytogenes* was detected on uterine swabs had histopathologic evidence of either acute chorioamnionitis or intrauterine fetal infection. In one case, *L. monocytogenes* was cultured from the vaginal swab, placental histopathology demonstrated chorioamnionitis, and the infant had culture-proven listeriosis. The other 2 cases had symptoms consistent with listeriosis, positive listeria cultures in the newborns, and pathologic findings of acute chorioamnionitis (Table 2). None of the mothers had central nervous system (CNS) involvement and all recovered fully after delivery.

**Table 3.** Characteristics of 8 Maternal Cases of Listeriosis

No.	Age (y)	Gestation (wks)	Symptom Duration	Presentation	Culture Sites	Initial Antibiotic	Switch Antibiotic	Maternal Complications	Maternal Outcome	Fetal Outcome
19	32	18.9	1 wk	Fever (T_max_ 39.5°C), chills, headache, dysuria, WBC 12 × 10^9^/L	Blood	Ceftriaxone→ cefmetazole + clarithromycin	Amoxicillin/ clavulanate	Pyelonephritis	Recovered	C-section 5 mo later, healthy baby
34	33	23	1 d	Fever (T_max_ 39.6°C), diarrhea, WBC 24 × 10^9^/L	Blood	Ceftriaxone + metronidazole	None	None	Recovered	Fetal death; placental pathology:acute chorioamnionitis
6	30	26.7	2 d	Fever (T_max_ 39.4°C), abdominal pain, vaginal bleeding, WBC 28 × 10^9^/L	Uterine swab	Cefuroxime + metronidazole	No change	Late abortion	Recovered	Fetal death; placental pathology:chorioamnionitis.
23	31	31	3 d	Ingestion of roasted lamb and rabbit in a Mongolian village 5 d before, decreased fetal movement 3 d, fever (T_max_ 39°C) 1 d, diarrhea, abdominal pain, WBC 19 × 10^9^/L	Uterine swab	NA	NA	None	Recovered, C-section (severe meconium stained amniotic fluid)	Infant listeriosis (case no. 24,Table 2); placental pathology: acute chorioamnionitis
15	28	37.1	1 d	Fever (T_max_ 39°C), WBC 15 × 10^9^/L	Uterine swab	Ceftriaxone + metronidazole	Ampicillin + metronidazole	None	Recovered. C-section (meconium stained amniotic fluid)	Infant listeriosis (case no. 16,Table 2); placental pathology:chorioamnionitis
35	29	39.9	4 h	Fever (T_max_ 38.8°C) for 4 hours, decreased fetal movement for 1 d, WBC 9.29 × 10^9^/L	Vaginal swab	Ceftriaxone + metronidazole	No change	Intrauterine fetal hypoxia	Recovered, C-section (severe meconium stained amniotic fluid)	Infant listeriosis (case no. 36,Table 2); Placental pathology:chorioamnionitis
37	26	32.7	2 wk	Headache, fever (T_max_ 39.8°C) 2 wk, decreased fetal movement 1 wk, severe abdominal pain 1 d	NA	NA	NA	Infant listeriosis	Recovered, postpartum uterine curettage for retention of fetal membranes	Infant listeriosis (case no. 25,Table 2); placental pathology: NA
38	30	27	1 d	Sudden onset of lower abdominal pain	NA	NA	NA	Premature labor	Recovered	Late abortion, fetal death (case no. 28, Table 2); placental pathology: acute chorioamnionitis

Abbreviations: C-section, cesarean section; NA, not available; T_max_, maximal temperature; WBC, peripheral white blood cell count.

Obstetrical outcomes included 5 cases of listeriosis in the infants postpartum. All 5 cases were the result of listeria infections during the third trimester of gestation, and a single one of these cases was fatal. There were 2 induced/late abortions as a result of listeria infections during the second trimester of gestation, and a normal pregnancy outcome for a single second-trimester infection.

### Nonmaternal Listeriosis

Among the 25 nonmaternal cases, the median age was 47 years (range, 18–79 years), and 72% (95% CI, 52.5%–85.7%) were female. Twenty-three (92%; 95% CI, 75.03%–97.78%) infections occurred in patients with significant comorbidities (Table 4). Ten (40%) patients had concurrent neoplasms: 2 cases each of leukemia, multiple myeloma, liver cancer, and rectal cancer, and 1 case each of breast cancer and abdominal malignant metastases from an unknown primary. Ten nonmaternal infections occurred in patients with autoimmune diseases: 6 cases in patients with systemic lupus erythematosus (SLE), 2 cases in patients with dermatomyositis, 1 case in a patient with Still's disease, and 1 in a patient with mixed connective tissue disease. Other comorbidities included diabetes mellitus and polycystic kidney disease with chronic renal failure. Ten (40%) nonmaternal adult listeriosis cases were receiving chronic corticosteroids at the onset of symptoms, and 6 (24%) had received chemotherapy within 2 months before the onset of listeriosis.

**Table 4.** Characteristics of 25 Cases of Nonmaternal Listeriosis

No.	Sex	Age (y)	Comorbidities	Predisposing Factor	Healthcare-Associated	Duration	Presentation	Culture Sites	Complications	Outcome
31	F	24	Acute lymphoblastic leukemia (L2)	Chemotherapy, neutropenia	Yes	1 d	Abdominal pain × 2 wk before admission, sudden fever (T_max_ 40.1°C) hospital day 12	Blood, CSF	Sepsis (*Listeria*, *E. coli*), cerebral hemorrhage, coma	Death hospital day 25
3	F	43	Metastatic liver disease; unknown primary	Neoplasm	No	3 d	Intermittent abdominal pain for 1 mo, fever (T_max_ 39.3°C), and headache 3 d	Blood, CSF	Meningitis, coma	Death hospital day 6
1	M	53	Multiple myeloma	Chemotherapy, chronic use of melphalan, thalidomide	No	2 d	Fever (T_max_ 40.7°C), headache, loss of consciousness	Blood, CSF	Septic shock, meningitis, ARF, GI perforation	Death hospital day 8
4	M	20	None	None	No	3 wk	Sudden onset fever (T_max_ 40°C), headache, worsening mental status (delirium, coma), ventricular enlargement, placement of external CSF shunt, intubated	CSF, sputum	Meningo-encephalitis, pneumonia, MOF, coma, central diabetes insipidus	Death hospital day 12
18	F	47	Dermatomyositis	Prednisone 40 mg/d	No	3 d	Fever, dizziness, and dysphagia, sudden cyanosis and coma while in emergency room	Blood, CSF	Meningitis, HAP (MRSA, *Enterobacter*) brain stem stroke, brain death	Death hospital day 20
2	F	56	SLE and abdominal malignancy of unclear primary	Prednisone 30–40 mg/d, CTX 0.4/wk	Yes	3 d	Admitted with fatigue, edema, and jaundice. Fever (T_max_ 38.5°C) started 3 d after admission.	Blood	Pneumonia, bacterial sepsis, MOF	Death hospital day 25
14	F	23	SLE	Prednisone 50–80 mg/d	No	1 d	Fever (T_max_ 39.2°C), epigastric pain for 1 d, epistaxis	Blood	Acute liver failure hepatic encephalopathy, coma, GI bleed, respiratory failure	Death hospital day 7
21	M	71	Rectal cancer, hepatic metastases	Chemotherapy	No	1 d	Fever (T_max_ 40°C) after chemotherapy, stool OB(+)	Blood	Coma, seizure, septic shock	Death hospital day 4
27	F	33	SLE with nephropathy	Prednisone 60 mg/d, 2 course of MP pulses	Yes	2 mo	Diarrhea and abdominal pain for 2 mo; sudden onset fever (T_max_ 39.2°C) on day 26 after admission	Blood	Multiple hospital-acquired infections, septic shock	Death hospital day 30
5	F	43	Dermatomyositis, DM, HCC	Prednisone 80 mg/d, CTX 0.4/wk	Yes	4 d	Fever (T_max_ 39.7°C) started on day 20 after admission, with headache, left hemiplegia	Blood	Sepsis (meningitis)	Recovered
8	F	22	SLE with nephropathy	Prednisone 50–60 mg/d, 2 courses of MP pulses + hydroxychloroquine 0.2 bid + CyA/Dapsone/MMF	Yes	2 wk	Fever (T_max_ 40°C) and diarrhea started on day 40 after admission	Blood	Meningitis	Recovered
10	M	53	Still's disease	Prednisone 50–60 mg/d or dexamethasone 5 mg/d, methotrexate 15 mg/d	Yes	1 d	Fever (T_max_ 39.6°C) started on day 44 after admission, with headache, vomiting, change in mental status	Blood, CSF	Meningitis, respiratory failure, MRSA pneumonia	Recovered
7	F	18	SLE	None	No	2 wk	Fever (T_max_ 39°C) headache and vomiting for 2 wk, and diplopia 1 d	Blood, CSF	*Cryptococcus neoformans* also grew from blood cultures	Recovered
30	M	74	DM, chronic kidney disease	None	No	2 d	Fever (T_max_ 39.2°C), nausea, vomiting	Blood, CSF	Meningitis, HAP	Recovered
17	M	69	None	None	No	4 d	Fever (T_max_ 39°C), change in mental status	CSF	Coma, ARF, pneumonia	Recovered
13	F	53	SLE with nephropathy	Prednisone 30 mg/d, CTX 0.4/wk	No	2 d	Fever (T_max_ 39.4°C), headache, vomiting, loss of consciousness	CSF	Meningitis	Recovered
12	F	45	Mixed connective tissue disease	Prednisone 60 mg/d	No	6 d	Fever (38.5°C), headache and altered mental status	CSF	Meningitis, DVT	Recovered
20	F	60	Non-Hodgkin lymphoma and lymphoblastic leukemia	Chemotherapy and neutropenia	Yes	5 d	Fever (T_max_ 40°C) started 5 d after chemotherapy on hospital day 9	Blood	Perianal abscess	Recovered
26	F	42	Breast cancer with metastases to bone, liver, and lungs	Neratinib (HKI-272), neutropenia	Yes	3 d	Fever (T_max_ 39.8°C), oral ulcers, diarrhea, after HKI-272 on hospital day 15	Blood	None	Recovered
11	F	36	Ulcerative colitis, hepatic cirrhosis, AIH	Prednisone 40 mg/d	Yes	1 d	Fever (T_max_ 39.7°C) and hepatitis on hospital day 21	Blood	None	Recovered
9	F	49	DM	None	No	5 d	Fever (T_max_ 40.5°C) abdominal bloating, headaches	Blood	Urosepsis	Recovered
22	M	79	Polycystic kidney disease, CRF	None	No	4 d	Fever (T_max_ 39°C), left upper quadrant abdominal pain	Blood	None	Recovered
32	M	59	Ulcerative colitis, rectal cancer with diffuse metastases	Chemotherapy	Yes	1 d	Fever (T_max_ 40°C) on hospital day 24	Blood	Candidiasis	Recovered
33	F	36	Multiple myeloma	None	Yes	1 d	Fever (T_max_ 38°C), on hospital day 3	Blood	HAP	Recovered
29	M	59	DM	None	No	2 d	Fever (T_max_ 39.9°C), diarrhea, abdominal pain, mental status change	Blood, CSF	None	Recovered

Abbreviations: AIH, autoimmune hepatitis; ARF, acute renal failure; bid, twice daily; CRF, chronic renal failure; CSF, cerebrospinal fluid; CTX, cyclophosphamide; CyA, cyclosporine A; DM, diabetes mellitus; DVT, deep vein thrombosis; *E. coli*, *Escherichia coli*; GI, gastrointestinal tract; HAP, hospital-acquired pneumonia; HCC, hepatocellular carcinoma; MMF, mycophenolate mofetil; MOF, multiple organ failure; MP, methylprednisolone; MRSA, methicillin-resistant *Staphylococcus aureus*; OB, occult blood; SLE, systemic lupus erythematosus; T_max_, maximal temperature.

Fever (96%), CNS involvement (64%), and gastrointestinal symptoms (48%) were the most common presentations. *Listeria monocytogenes* was cultured from blood (n = 13), blood and CSF (n = 8), CSF (n = 3), and CSF and sputum (n = 1).

The 2 cases of *L. monocytogenes* that occurred in otherwise healthy hosts had early CNS involvement, manifested by coma. The first, a 20-year-old patient, experienced sudden onset of diarrhea, fever, and headache and deteriorated rapidly. He was intubated and treated at a local outside hospital first (where no *L. monocytogenes* was isolated from his cultures), and *L. monocytogenes* was isolated from sputum and CSF 4 weeks after the onset of gastrointestinal symptoms (Table 4, patient 4). The second, a 69-year-old previously healthy man, developed sudden fever and convulsions (Table 4, patient 17) rapidly progressing to coma complicated by acute renal failure and pneumonia. His condition improved after an extensive hospital stay and he was transferred to an outside institution for further rehabilitation. No long-term follow-up was available.

Seventy-two percent of adults were treated empirically with cephalosporins and all were switched to ampicillin after the positive culture results became known. Among the 9 (36%; 95% CI, 20.25%–55.48%) fatal cases, 8 had severe underlying diseases and developed complications after being infected with *L. monocytogenes*. All died of multiple severe complications within 30 days after the onset of infection. The fatal cases were more likely to have sepsis (n = 9), rapid onset of coma (n = 6), and multiorgan failure (n = 3).

### Healthcare-Associated Listeriosis

**Figure 1.**
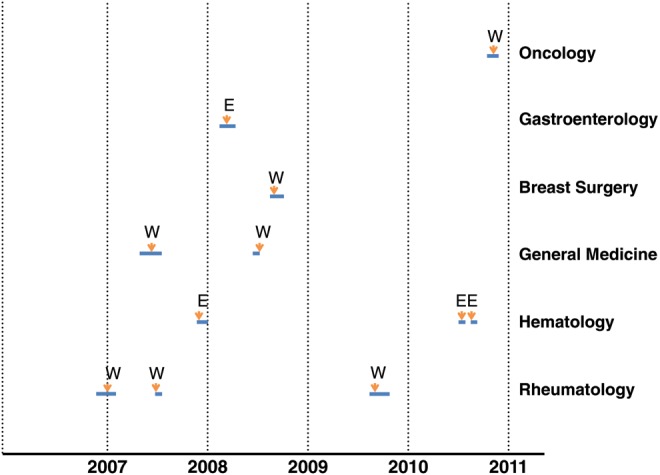
Distribution of admission departments and calendar years for 11 healthcare-associated cases of listeriosis. Admission duration is shown in the blue lines in proportion to the time period, and the onset of symptoms consistent with listeriosis is indicated with yellow arrows The letters E and W represent the eastern and western campuses of Peking Union Medical College Hospital.

Eleven (44%; 95% CI, 26.67%–62.93%) nonmaternal adult cases were healthcare-associated. The patients were admitted for treatment of rheumatologic diseases (n = 6), malignancy (n = 4), and malignancy with ulcerative colitis (n = 1). The admitting department and its location, timing of infection, and duration are illustrated in Figure 1. The onset of symptoms related to listeriosis occurred after a median of 20 days (range, 3–44 days) following admission. The mortality among healthcare-associated cases was 27.2% (95% CI, 9.74%–56.56%).

These infections were first detected in 2006, and there were 1, 3, 3, 1, and 3 infections detected per year from 2006 to 2010, consecutively. These infections were scattered in 6 different wards, both in the eastern and western campuses of PUMCH. There were 3 cases each in the rheumatologic and hematologic wards and 2 cases in the general medicine ward. Only 2 cases appeared to be clustered in space and time. Nine of these 11 cases did not appear to be clustered. There was no consistent pattern (location, seasonality, and timing) that emerged for the 9 nonclustered cases. The source of their infection could not be determined.

## DISCUSSION

The most striking finding from this case series is the prevalence of nonclustered healthcare-associated cases of listeriosis. Eleven of 25 nonmaternal listeriosis cases were healthcare-associated. These infections did not appear to be clustered in time and space. There are rare reports of healthcare-associated transmission of *L. monocytogenes* via contaminated foods, healthcare workers, and infected patients, but most of these were clustered in time and space. For example, a recent study reported a cluster of 6 *L. monocytogenes* infections in hospitalized adults during a 10-month period in Brazil [28]. The median age of these patients was 80 years and all had underlying severe comorbidities. Four isolates belonged to a single pulsed-field gel electrophoresis (PFGE) genotype, suggesting a common source. The epidemiological investigation pointed to the hospital kitchen as the possible source of contamination.

It is intriguing to speculate whether these healthcare-associated cases were the result of in-hospital acquisition, or whether this was the result of colonization. Until this retrospective case series was conducted, we had absolutely no insight about the frequency of these healthcare-associated cases. The cases were not clustered in time or space so they did not elicit additional surveillance. Although we could not perform PFGE on the specimens from our study, the majority did not cluster in time or space, suggesting that a common source was unlikely. Investigators have recognized for >20 years that *L. monocytogenes* can be carried in the gastrointestinal tract [29–31]. *Listeria monocytogenes* can be isolated in the stool of 1%–10% of the population, where it can persist without causing symptoms [32]. Using repeated sampling, *Listeria* can be detected in the feces of nearly 70% of healthy nonpregnant individuals and 44% of pregnant women [21, 31]. MacGowan et al found that *Listeria* was isolated from 5.6% (10/177) of renal transplant recipients on 1 or more occasions over the period of a year; moreover, >1 species or serovar of listeria can be isolated from 40% of fecal carriers, and no cases of clinical infection occurred in any fecal carriers [33]. Fecal, cervicovaginal, and oropharyngeal carriage of *L. monocytogenes* has been reported as a possible predisposing factor for perinatal listeriosis [34, 35]. In one study conducted by Schuchat et al [36], asymptomatic carriage of the illness-associated strain of *L. monocytogenes* was identified in nearly one-fifth of household contacts of patients with sporadic listeriosis, and no cases of secondary disease were detected within households in this study. Their findings suggest that gastrointestinal carriage of pathogenic strains of *L. monocytogenes* is not uncommon in contacts of cases, underscoring the critical role that host susceptibility plays in determining whether illness occurs following exposure to this organism. All of our cases of healthcare-associated listeriosis had severe underlying immunosuppression. Besides immunosuppression, many of our patients had underlying diseases involving the gastrointestinal tract, or their therapy could impact the integrity of the intestinal mucosa. So, the role that gastrointestinal colonization of *Listeria* played in the pathogenesis of these healthcare-associated infections warrants further study.

After the discovery of these nonclustered healthcare-associated cases, we have implemented a more aggressive approach: all healthcare-associated cases will be thoroughly investigated for both prehospital and in-hospital exposures. We are also saving all bacterial isolates for DNA fingerprinting. This more aggressive approach may help us better define the source of these infections.

Among the healthcare-associated listeriosis cases, one patient with diffuse metastatic breast cancer experienced sudden onset of fever, oral ulcers, and diarrhea after 3 days of HKI-272 treatment (Table 4, patient 26). Blood culture yielded *L. monocytogenes*. The HKI-272 therapy was discontinued and antibiotic treatment was initiated, and the patient fully recovered. HKI-272, also known as neratinib [37], is an oral, irreversible dual EGFR/HER2 inhibitor for breast and non-small-cell lung cancer. Phase 1 and 2 studies reported gastrointestinal adverse events, including diarrhea (89%), nausea (29%–64%), and vomiting (23%–50%). Approximately 30% of patients required discontinuation or dose reduction due to severe diarrhea. Cases of listeriosis were reported among patients undergoing therapy with other biologic agents such as infliximab (antitumor necrosis factor agents) [38–41], etanercept (a tumor necrosis factor antagonist) [42], and trastuzumab (a monoclonal antibody against the HER2 receptor) [43].

Forty percent of our cases had underlying rheumatologic diseases. This proportion is higher than what was previously reported in the literature [38]. Although PUMCH does not specifically specialize in the treatment of rheumatic diseases, we do have a large population of such patients. Persons of Asian descent have a higher incidence of SLE, compared with other races [44–46]. Given the paucity of published reports on *L. monocytogenes* from East Asia, this may explain the higher incidence among patients with rheumatic diseases in our report. This may also have impacted the sex distribution of cases. Traditionally*, L. monocytogenes* has been reported more often among men than women. The male to female ratio in our study was 1:1.8. This may reflect the increased predisposition of rheumatic diseases among women [47–49].

Comorbidity plays a very important role in the prognosis of listeriosis [18]. Eighty-one percent of 225 patients with listeriosis studied in France had a predisposing immunocompromising condition, whose severity was the major prognostic factor [17]. In our population, 92% of nonmaternal listeriosis cases were immunosuppressed.

Our cases of infant listeriosis mirrored the cases reported in the literature, as did their outcomes. We did not observe any late-onset cases of infant listeriosis, as reported by other authors [9, 22, 50–52]. Similarly, the characteristics of our maternal listeriosis were similar to those reported in the literature.

This study has several limitations. First, it is a retrospective assessment over a protracted timespan. As such, we were unable to obtain specimens for molecular testing, and we were unable to clarify additional issues relating to certain in-hospital epidemiological exposures. Second, it consists of a relatively small sample size, and our findings may not be necessarily generalizable to other populations or settings. Third, cases of listeriosis in China are not routinely reported to public health authorities. As such, the epidemiology of listeria is not well defined. Our case series reflects a selection bias toward hospitalized (ie, sicker) patients and may not reflect the overall epidemiology of listeria.

Nonclustered healthcare-associated cases of *L. monocytogenes* occurred at a large tertiary care hospital in Beijing, China. The source of these infections is unclear. Although rare, in the setting of immunosuppression, *Listeria* should be considered in the differential diagnosis of healthcare-associated infections—even in the absence of a point-source outbreak.
